# Psychometric analysis of the flow short scale translated to Finnish

**DOI:** 10.1038/s41598-022-24715-3

**Published:** 2022-11-22

**Authors:** Michael Laakasuo, Jussi Palomäki, Sami Abuhamdeh, Otto Lappi, Benjamin Ultan Cowley

**Affiliations:** 1 Cognitive Science, Department of Digital Humanities, Faculty of Arts, University of Helsinki, Siltavuorenpenger 1 A, 00014 Helsinki, Finland; 2 Quality of Life Research Center, Claremont, USA; 3 Traffic Research Unit, University of Helsinki, Helsinki, Finland; 4 Faculty of Educational Sciences, University of Helsinki, Helsinki, Finland

**Keywords:** Psychology, Human behaviour

## Abstract

Flow is a well-known construct describing the experience of deep absorption in a task, typically demanding but intrinsically motivating, and conducted with high skill. Flow is operationalized by self-report, and various instruments have been developed for this, but none have been made available in the Finnish language in thoroughly validated form. We present a psychometric scale-validation study for the Finnish translation of the Flow Short Scale (FSS). We collected data from 201 Finnish speaking participants using the Prolific Academic platform. We assessed the scale’s factorial structure using Mokken scale analysis, Parallel Analysis, Very Simple Structures analysis and a standard Confirmatory Factor Analysis. We then evaluated how correlated was the FSS with the Flow State Scale and Flow Core Scale. Finally, we evaluated how well the FSS distinguished Flow-inducing experiences from boring (non-Flow-inducing) experiences. Taken together, our results show that an 8-item, two-factor version of the scale was a justified instrument with good psychometric properties.

## Introduction

Why do people perform time-consuming, difficult, and sometimes even dangerous activities for which they receive no discernible extrinsic rewards? Half a century ago, this question prompted a program of research that involved extensive interviews with hundreds of rock climbers, chess players, athletes, and artists^1^. In all the groups studied, the respondents reported a very similar subjective experience, a state of deep absorption in moment-to-moment activity that was accompanied by a sense of control and diminished self-consciousness. This ‘optimal experience’ was eventually called “Flow”, because in describing it, several respondents used the metaphor of a current carrying them along effortlessly^1^.

Since the concept’s introduction in 1975, interest in Flow has grown considerably. Today the Flow experience is the focus of hundreds of empirical studies from a diversity of fields including educational psychology, recreation and leisure sciences, game design, and many others^2,3^. One reason for this interest is undoubtedly the large number of positive outcomes associated with Flow, including heightened creativity (e.g.^4^), enhanced learning (e.g.^5^), and peak performance in sports (e.g.^6^). Furthermore, because Flow typically occurs when an individual engages in an activity which stretches his or her existing capacities, the intrinsically rewarding (i.e. ‘autotelic’) nature of Flow has positive implications for skill development and personal growth^7^.

Despite the many good reasons for researching Flow, anyone who wishes to do so faces a methodological challenge in how to operationalize the construct. Though many Flow self-report measures exist, no single standard has emerged in the field. Indeed, in a recent review of Flow operationalizations found in the Flow literature, across the 42 reviewed studies (from 2014-’19), Flow was operationalized in 24 distinct ways^8^. A second issue faced by many researchers who wish to investigate Flow is the limited number of scales that have been re-validated for use in languages other than English (see literature review below). Such revalidation studies are crucial, because one cannot assume that a scale developed in one language will perform similarly when adapted to another language. Thus, pending an international consensus study to develop a universal multilingual Flow instrument, the most parsimonious way to operationalize Flow for a given language context is to translate and revalidate an existing, popular Flow scale^9,10^.

The contribution of the current study is twofold: *first*, we translate and revalidate an existing Flow scale for the Finnish language; *second*, by re-examining the factor structure of the scale, we provide insight into the generalizeability of the original scale across languages and contexts. Existence of a validated short Flow scale is valuable for Finnish research, since there are a wealth of human-focused studies in relevant areas, such as education, music, games and gambling, sports, and traffic psychology.

*Examining the state of the art*, Abuhamdeh’s^8^ review indicates that the two most popular scales for measuring the experience of Flow are The Flow State Scale^11^ (which measures flow in the context of physical activities) and (to a lesser extent) the Flow Short Scale^12^ (which is intended as a more general purpose scale). Although the Flow Core Scale is used much less frequently, it distinguishes itself from most other flow scales by only including items reflecting the flow experience itself (rather than conditions proposed to elicit flow such as optimal challenge) and thus appears to have particularly high face validity^8^. Revalidations of some of these scales have been published to adapt them to specialist domains, such as reading^13^, web browsing^14^, and clinical populations^15^.

We conducted a novel and brief literature review to assess whether and how often these scales have been revalidated for other languages. We searched for each scale’s exact name on Scopus under Title, Abstract, and Keywords—we obtained 82, 20, and zero hits for State, Short, and Core scales respectively. Examining the results of this search suggests that there have been no validation studies of the Core scale in new languages. For the Flow State scale, we found *domain-specific* revalidated translations in athletics (Turkish^16^; Brasilian Portuguese^17^; Spanish^18^; French^19^; Greek^20^), music performance (Spanish^21^—notably, this study found issues with the item related to temporal effects in Flow, as did we), and occupational therapy (Japanese^22^). The Flow State scale has also been revalidated in a Japanese translation for general-purpose use^23^, while the *Dispositional* Flow trait scale has been translated and validated for general use in Italian^24^.

For the Flow Short Scale^12^ we found only one revalidation, which examined the factor structure of a Greek version of the scale^25^. In that study, the final scale structure was notably different from the one found in the original validation study^26^. This result serves to underscore the importance of examining the performance of the Flow Short Scale in both different languages and different contexts.

*The Flow Short Scale* (hereafter *FSS*; originally published in German by^26^, first in English by^12^), designed to measure state-level Flow, was chosen here for translation and revalidation in Finnish based on two primary considerations. First, because Flow is often measured in the context of repeated measures designs, we chose a short scale, that would minimize participant burden. Conveniently, the FSS consists of only ten items (typically accompanied by three items to tap perceived importance). Second, because we worked under the assumption that others may subsequently use our derived scale, we chose a scale which was relatively popular among Flow researchers. FSS has indeed been used to assess Flow in the context of a wide range of sports, games, and other goal-directed activities^27–30^. As indicated above^8^, the Flow State Scale^11^ is the more popular of the two most commonly used scales to measure state-level Flow. However, we preferred the FSS because (1) the FSS conflates the experience of Flow and the conditions of Flow to a lesser degree than the Flow State Scale, and (2) the FSS is shorter than the Flow State Scale.

In summary, our experiments and analyses, detailed below, together suggest that an 8-item, two-factor Finnish FSS—that excludes items 1 and 3 from the original scale—is a justified, validated psychometric instrument.

## Method

The Finnish FSS was first used as the dependent variable (DV) in previous studies on the relationship between Flow and performance, conducted by the authors^31–33^. We subsequently determined the need (outlined above) to fully validate the translated scale with a separate study designed for scale validation (that is, larger sample size and no performance task). We obtained such data via the Prolific Academic online platform. We performed a series of psychometric analyses focused on the FSS and its factorial structure (Mokken scale analysis, Parallel Analysis, Very Simple Structures analysis and Confirmatory Factor Analysis). Then, we performed a sensitivity analysis evaluating the correlations between FSS and two other well-known Flow state scales, the Flow State Scale^11^ and Flow Core Scale^34^. Finally, we evaluated the FSS’s ability to discriminate between self-defined ‘Flow-inducing’ and ‘boring’ experiences.

### Participants and design

In total, 201 Finnish speaking individuals (94 female, mean age = 29.2) participated in a cross-sectional study on the commercial online platform Prolific Academic. Of the participants, 89 (44.5 %) had at least a bachelor’s degree. Participants were compensated $\pounds$1.5 pounds sterling (about 1.76 euros), and participation took 15.2 min on average.

The current study was run as a part of a larger project^33^, which was approved by the University of Helsinki Ethical review board in humanities and social and behavioral sciences (statement 31/2017; study title MulSimCoLab). Under Finnish law, this particular experiment did not require ethical permission as it was non-invasive, non-sensitive research conducted on adults. The experiment was carried out in accordance with the code of ethics of the world medical association (Declaration of Helsinki) for experiments involving humans. Informed consent was obtained from all participants.

### Procedure

The design was cross-sectional. Participants first gave informed consent and then completed all psychometric measures in the same order. Before the measures, participants read instructions telling them to spend 1 min to recall and visualize a previously experienced Flow-inducing event (as per^11^), and were then asked to write down a short description (max 10 words) of that event in a text box.

On the next pages the participants were instructed to fill in the Flow Short Scale, Flow Core Scale, and Flow State Scale in that order, based on their memory of the Flow-inducing event. After filling in the scales, participants were instructed to recall and visualize a boring, non-Flow-inducing, event and again fill in the Flow Short Scale, but this time based on their memory of the boring event (translations below). As before, participants were also asked to give a short 10-word description of their (boring) experience. Finally, all participants were debriefed, thanked and dismissed.

Before filling in the FSS for the first time, participants received the following instructions (translated from Finnish) to recall a ‘Flow-like experience’:

*“After about 1.5 min, a button will show up at the bottom of the screen—click on it to continue. On the next page you will be asked questions regarding a small exercise we would like you to do. Use this short waiting time to recall a successful experience that required some skill but happened fluently, and in which you absorbed in (an experience where you felt like in a ‘Flow’ state). It can be a positive experience of rock climbing, playing the guitar, meditating, or a successful motocross track—it can be anything. It doesn’t matter what the experience or situation was, as long as it was a ‘Flow’ experience to you, yet also challenging. However, it is important that you concentrate on imagining this experience vividly.”*

Before filling in the FSS for the second time, participants received the following instructions to recall a ‘boring experience’ (translated from Finnish):

*“Now we would like you to repeat the previous exercise, but this time we ask you to recall a situation that has been particularly dull, perhaps difficult and boring, and felt like it lasted forever (a situation where you definitely did not experience a ‘Flow’ state). It can be, for example, a particularly unexciting day at a summer job at an assembly line, or a dragging and tiresome cleaning day, or a mechanical and repetitive task, but one that is challenging enough to keep you from focusing even on your own thoughts. It doesn’t matter what the experience or situation was, as long as it was particularly dull and boring. However, it is important that you concentrate on imagining this experience vividly.”*

### Materials

#### Flow short scale (FSS)

The FSS^12^ uses a 10-item scale to tap Flow, composed of two sub-scales—one intended to measure ‘absorption’ (4 items; e.g. “I do not notice time passing”), and the other intended to measure ‘fluency’ of performance (6 items; e.g. “My thoughts/activities run fluidly and smoothly”). Items 1, 3, 6 and 10 form the Absorption subscale in FSS according to the original scale structure^12^, while remaining items form the Fluency subscale. The scale is evaluated on a 7-point Likert scale with anchors varying from ‘Not at all’ to ‘Very much’. An additional 3 item measure of perceived importance is administered with the FSS to determine the experienced importance of the given task (e.g. “I must not make any mistakes here”). The FSS has been made publicly available under Creative Commons Share Alike 3.0 License, which permits the scale to be shared and adapted for any purpose, see http://www.psych.uni-potsdam.de/people/rheinberg/messverfahren/fks1-e.html.

The FSS items were first translated from English to Finnish for use in the study by^31^, modified slightly to reflect that study’s game-like experimental task. Two of that paper’s authors (native Finnish speakers, advanced English qualifications (e.g. International Baccalaureate), no formal qualifications for English-Finnish translation) first made translations independently; these translations were compared and revised, then reviewed by other Finnish-native authors, and revised.

In the validation study reported here, the activity to be reflected on could be anything (unlike in^31^), so the items referring to ‘playing’ were changed minimally to refer to ‘activity’ (e.g., item 10 [“Syvennyin peliin täysin”/“I delved into the game fully”] was changed into [“Syvennyin toimintaan täysin”/“I delved into the activity fully”]). Note that in the original item 2, the words ‘fluidly’ and ‘smoothly’ are almost synonymous, and in a gaming context, they are aptly captured by the single word ‘sujuvasti’ (which could also mean ‘fluently’).

The translations Table 1 shows the items in their original form (right column), the Finnish version (left column) translated from the original, and the English version translated from the Finnish (middle column).

**Table 1:** FSS items in original English form; translated to Finnish in general form for the validation study and game-specific form for^31^; and back-translated from game-specific form to English for verification.

Item	Finnish translation (general) $\leftarrow$	Original English
1	Toiminta tuntui juuri sopivan haastavalta	I feel just the right amount of challenge
2	Toimin sujuvasti	My thoughts/activities run fluidly and smoothly
3	En huomannut ajankulkua	I do not notice time passing
4	Pystyin hyvin keskittymään	I have no difficulty concentrating
5	Mieleni oli selkeä	My mind is completely clear
6	Uppouduin täysin toimintaani	I am totally absorbed in what I am doing
7	Löysin oikeat liikkeet kuin itsestään	The right thoughts/movements occur of their own accord
8	Olin koko ajan tilanteen tasalla	I know what I have to do each step of the way
9	Tunsin hallitsevani tilannetta	I feel that I have everything under control
10	Syvennyin toimintaan täysin	I am completely lost in thought
11	Koin toiminnassani onnistumisen tärkeäksi	Something important to me is at stake here
12	Minusta tuntui siltä, etten saisi tehdä yhtäkään virhettä	I must not make any mistakes here
13	Pelkäsin epäonnistuvani	I am worried about failing
	$$\downarrow$$	

Item	Finnish translation (game) $\rightarrow$	Back-translation
1	Peli tuntui juuri sopivan haastavalta	Playing the game, I felt just the right amount of challenge
2	Pelasin sujuvasti	I played fluently
3	En huomannut ajankulkua	I did not notice time passing
4	Pystyin hyvin keskittymään	I found it easy to concentrate
5	Mieleni oli selkeä	My mind was clear
6	Uppouduin täysin pelaamiseen	I immersed (myself) fully in playing
7	Löysin oikeat liikkeet kuin itsestään	I found the right moves spontaneously
8	Olin koko ajan tilanteen tasalla	I was able to cope with the task all the time
9	Tunsin hallitsevani tilannetta	I felt in control of the situation / I felt I had everything under control
10	Syvennyin peliin täysin	I delved into the game fully
11	Koin pelissä onnistumisen tärkeäksi	It was important to me to succeed in the game
12	Minusta tuntui siltä, etten saisi tehdä yhtäkään virhettä	I felt like I shouldn’t make any mistakes
13	Pelkäsin epäonnistuvani	I was worried about failing

#### Flow core sacale

The Flow Core Scale^34^ is a 10-item uniform scale intended to measure the state of being in Flow or ‘the zone’. Items are evaluated on a 7-point Likert scale from 1 (Strongly disagree) to 7 (Strongly agree). Example items are “I am *in the zone*” and “I am totally involved”.

#### Flow state scale

The Flow State Scale^11^ is a 36-item scale comprising 9 subscales with 4 items each. The subscales (example item in brackets) are: Challenge-skill balance (“I was challenged, but I believed my skills would allow me to meet the challenge”), Action-awareness merging (“I made the correct movements without thinking about trying to do so”), Clear goals (“I knew clearly what I wanted to do”), Unambiguous feedback (“It was really clear to me that I was doing well”), Concentration on task at hand (“My attention was focused entirely on what I was doing”), Paradox of control (“I felt in total control of what I was doing”), Loss of self-consciousness (“I was not concerned with what others may have been thinking of me”), Transformation of time (“Time seemed to alter (either slowed down or speeded up)”), and Autotelic experience (“I really enjoyed the experience”). Items are evaluated on a 7-point Likert scale from 1 (Strongly disagree) to 7 (Strongly agree).

### Analyses

All our analyses were conducted on the FSS items alone, excepting the external validity sensitivity analysis which compared FSS to the Flow State and Core scales. Analyses are presented in three categories: internal, external, and face validity. Most analyses are in the first category, because here we aim to address our second study purpose and thoroughly examine the internal structure of the scale.

We first ran a Mokken scale analysis on the FSS. Mokken scale analysis refers to a series of procedures where individual scale items and their properties are investigated in the context of the whole scale (we followed the guidelines of^35^). The analysis includes a number of distinct steps depending on the intended purpose and technical details of the scale. We present the results for: (1) homogeneity analysis for individual items (i.e. normed corrected item-scale covariance), (2) scalability analysis using the automated item selection procedure (to evaluate scale structure across multiple homogeneity index values), and (3) monotonicity analysis for individual items (i.e., do higher scores on individual items correspond to higher scores on the whole scale). We also performed Parallel- and Very Simple Structure—analyses^36–38^ to evaluate the factorial structure of the scale. Finally, based on the results of these analyses, we performed a standard Confirmatory Factor Analysis (CFA) using the Maximum Likelihood Satorra-Bentler correction estimation method (which is robust to violations of model assumptions, including non-normality and heteroscedasticity) on a two-factor solution of the FSS. Since the two factors are allowed to correlate freely, this analysis is analogous to an oblique rotation method in an exploratory factor analysis. Additional analyses are explained in situ below.

## Results

### Internal validity

The item-level homogeneity analysis is presented in Table 2. The recommended cut-off point for item homogeneity index values (also known as scalability coefficients) in the literature is 0.30^35^, which is not reached by items 1 and 3.

**Table 2:** Item-level homogeneity analysis for Flow Short Scale.

Item name	Homegeneity index	Standard error
FlowShort_1	**0.232**	0.051
FlowShort_2	0.404	0.038
FlowShort_3	**0.198**	0.045
FlowShort_4	0.472	0.033
FlowShort_5	0.396	0.039
FlowShort_6	0.412	0.039
FlowShort_7	0.391	0.038
FlowShort_8	0.436	0.037
FlowShort_9	0.421	0.038
FlowShort_10	0.419	0.038

Items with homogeneity index value < 0.30 are in bold.

The scalability analysis on different homogeneity index cut-off values is presented in Table 3. This analysis reveals how the average homogeneity of the whole scale changes when items load on different factors. In Table 3, zeros indicate unscalable items, a column of ones indicates a one-factor solution, and a column combining ones and twos indicates a two-factor solution for the whole scale. The analysis implies that, excluding items 1 and 3, a moderately scalable instrument can be formed from items 2, and 4–10. Higher scalability values seem to result in a theoretically-unsound scale structure. According to^35^, scalability index values should be at least 0.30, but preferably as high as possible. The FSS has acceptable scalability (between 0.35 and 0.45) when items 1 and 3 are dropped.

**Table 3:** Item selection for different levels of homogeneity index thresholds.

Item	Level of homogeneity															
	0.05	0.10	0.15	0.20	0.25	0.30	**0.35**	**0.40**	**0.45**	0.50	0.55	0.60	0.65	0.70	0.75	0.80
FlowShort_1	1	1	1	1	1	0	**0**	**0**	**0**	0	0	0	0	0	0	0
FlowShort_2	1	1	1	1	1	1	**1**	**1**	**1**	2	0	0	0	0	0	0
FlowShort_3	1	1	1	0	0	0	**0**	**0**	**0**	0	0	0	0	0	0	0
FlowShort_4	1	1	1	1	1	1	**1**	**1**	**1**	1	1	0	0	0	0	0
FlowShort_5	1	1	1	1	1	1	**1**	**1**	**1**	0	0	0	0	0	0	0
FlowShort_6	1	1	1	1	1	1	**1**	**1**	**1**	1	1	1	1	1	1	1
FlowShort_7	1	1	1	1	1	1	**1**	**1**	**0**	0	0	0	0	0	0	0
FlowShort_8	1	1	1	1	1	1	**1**	**1**	**1**	2	2	2	2	0	0	0
FlowShort_9	1	1	1	1	1	1	**1**	**1**	**1**	2	2	2	2	0	0	0
FlowShort_10	1	1	1	1	1	1	**1**	**1**	**1**	1	1	1	1	1	1	1

Bold columns show solutions with acceptable scalability, that retains a theoretically sensible scale structure.

**Figure 1:**
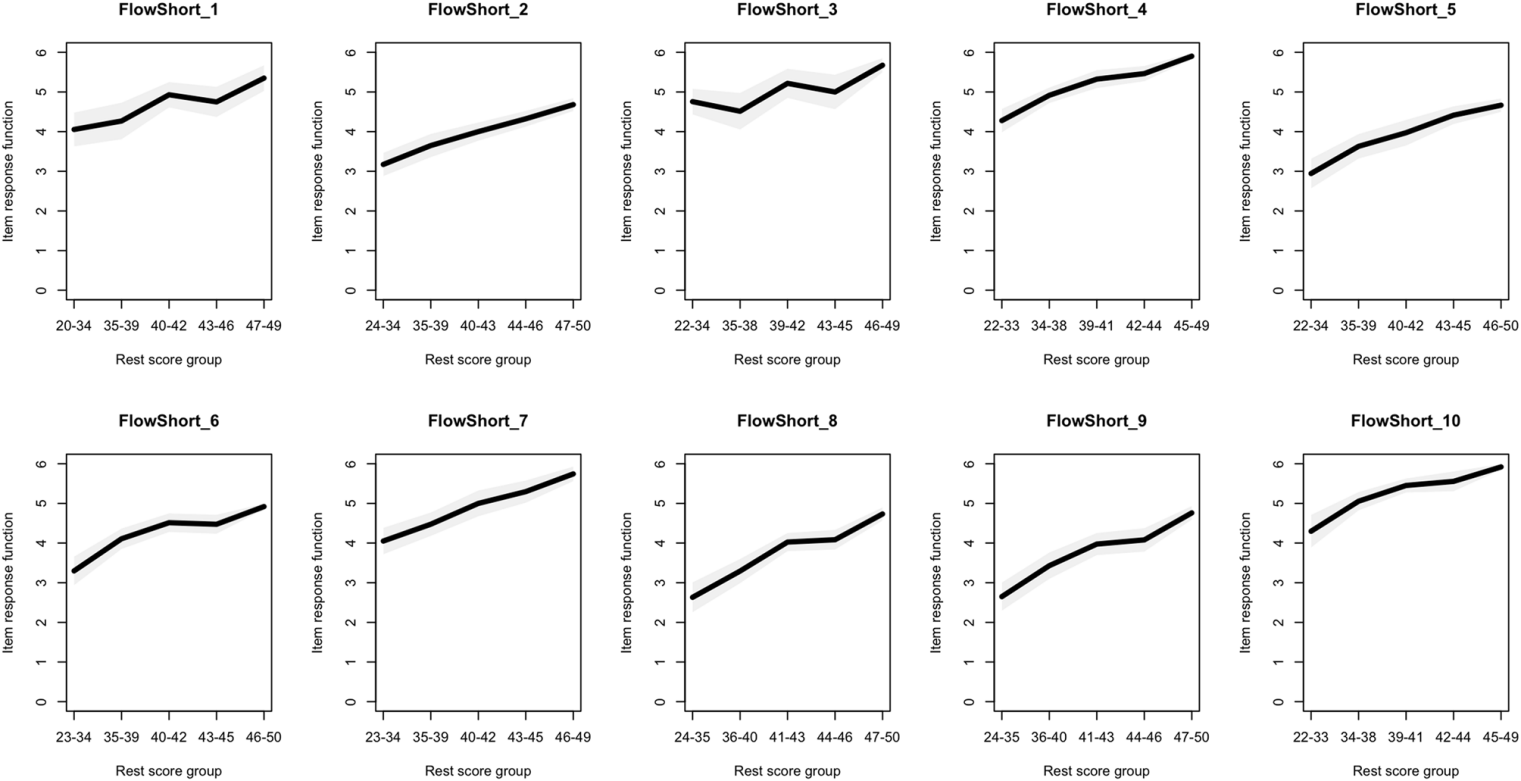
Monotonicity analysis of individual FSS items. The x-axis depicts the sum score of the whole scale. The y-axis depicts the item response function. Optimally, all item response functions should be relatively diagonal, or at the very least without downward dips. Items 1 and 3 have a clear zig-zag pattern, that is, a clear violation of monotonicity.

We then performed a monotonicity analysis. According to the monotonicity violation indices, two clear violators of monotonicity, at the scalability index value of 0.35, were items 1 and 3. Items 4, 6, 8 and 10 each had a single negligible monotonicity violation^37^. For graphical presentation of the monotonicity functions of individual items, see Fig. 1.

**Figure 2:**
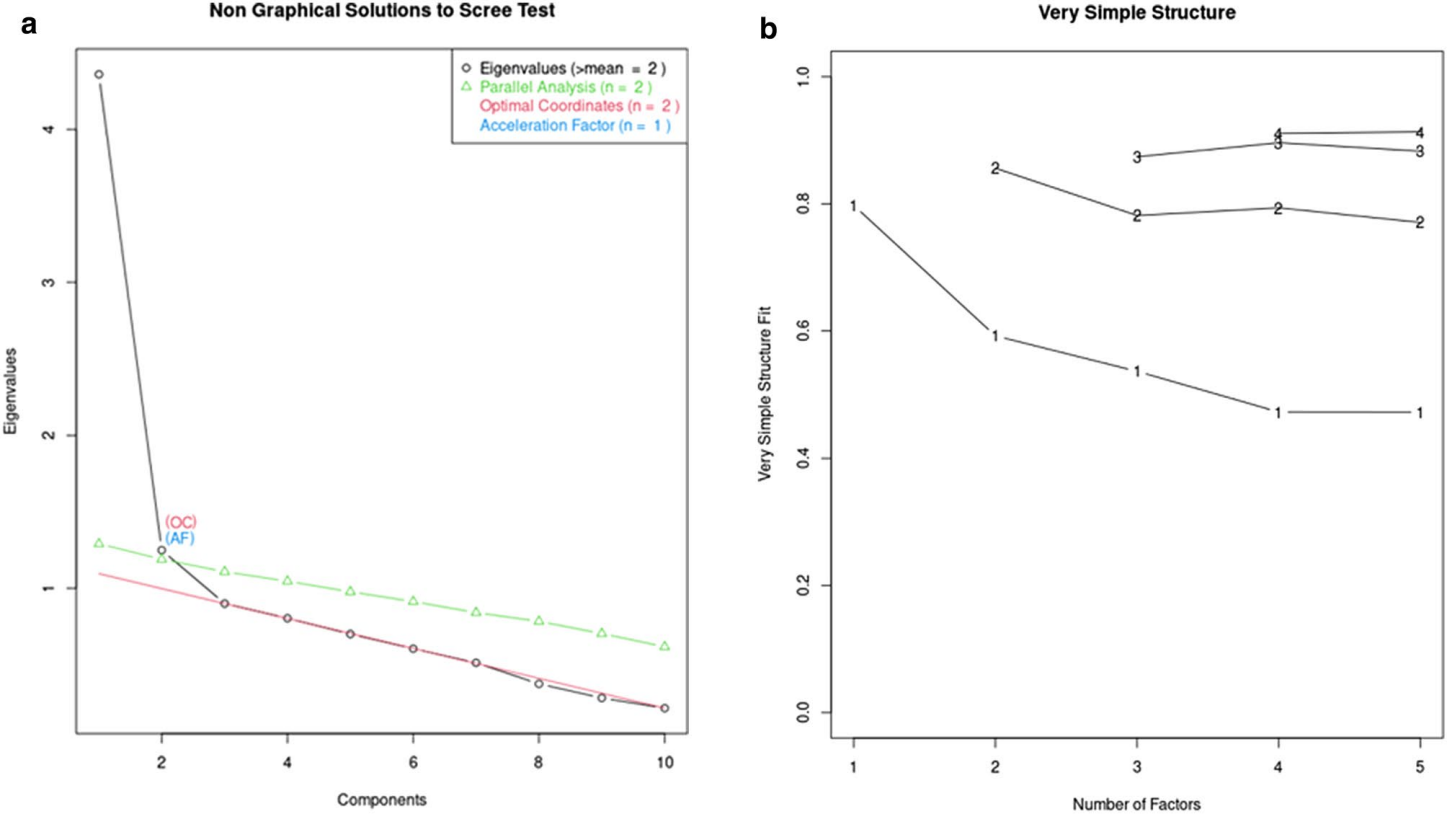
*Left*: Results of Parallel Analysis suggest that there are two factors contained in the 10 items that make the Flow Scale Short instrument. The green triangles represent random eigenvalues generated by the Parallel Analysis. The first two actually observed eigenvalues (black circles) were higher than the randomly generated eigenvalues (eigenvalue criterion). *Right*: VSS analysis suggest that the two-factor solution is optimal. Increasing factors beyond 2 results in increased BIC-values.

Next, we ran Parallel- and Very Simple Structure (VSS) analyses^36,37^, both of which suggested that the 10 FSS items contain two factors. In the VSS analysis, a two-factor solution has maximum complexity with a fit index of 0.86. Increasing the number of factors does not result in lower (i.e. better) Bayes Information Criterion values (the minimum of − 73.05 is reached with a two factor-solution; BIC-values in all other solutions were above − 73). See Fig. 2.

The analyses presented so far supports dropping items 1 and 3, since they (a) do not have the required level of homogeneity, and (b) violate the monotonicity assumption. In other words, items 1 and 3 do not pass the Mokken scale analysis. Furthermore, items 1, 3, 6 and 10 are conceptually part of a separate subscale in the FSS. Both Parallel Analysis and VSS analysis suggest that there are two factors in the scale. We therefore repeated the Mokken scale analyses by leaving out items 1 and 3. These new analyses still suggested that there are two factors in the scale, and that items 6 and 10 still had a minor tendency to violate the monotonicity assumption. Based on simply the eigenvalue criterion, Parallel Analysis suggested there might be two factors, while the VSS analysis suggested that there were either two or three factors.

**Figure 3:**
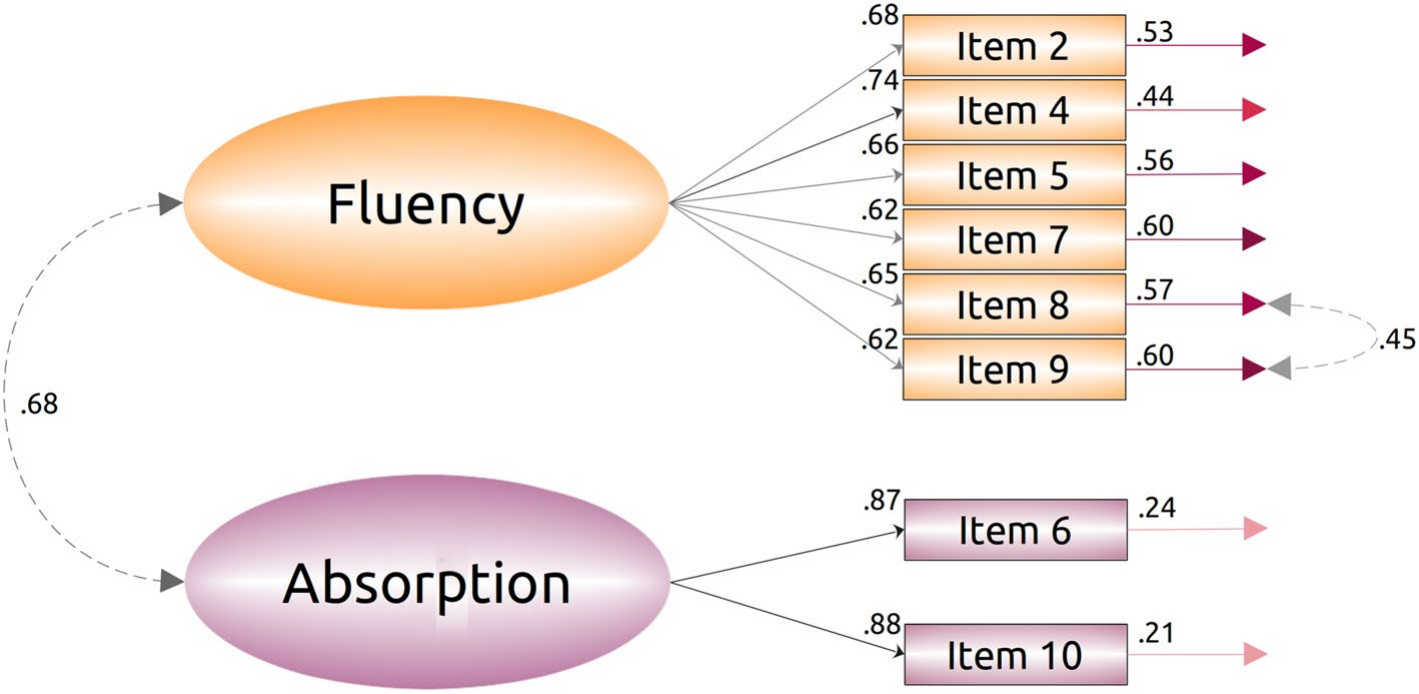
Final Confirmatory Factor Analysis for the 8-item Finnish FSS. This model has a good fit with the data: ${\chi }^2$(18) = 31.07, CFI = 0.97, TLI = 0.96, RMSEA = 0.06, 90% CI [0.027, 0.090], SRMR = 0.041.

Based on the results of the Mokken scale analysis, we ran a Confirmatory Factor Analysis (CFA), by allowing items 6 and 10 to load on a separate factor from the rest of the items (see Fig. 3 below). The resulting model had a relatively good fit with the data (${\chi }^2$(19) = 51.93, CFI = 0.94, TLI = 0.91, RMSEA = 0.093, 90% CI [0.06, 0.12], SRMR = 0.055). However, as an exploratory analysis, we added a single error correlation between the items 8 and 9, which had the highest modification index value (34.7). This substantially improved the scale (${\chi }^2$(18) = 31.07, CFI = 0.97, TLI = 0.96, RMSEA = 0.06, 90% CI [0.027, 0.090], SRMR = 0.041). We also fitted a single factor solution for the 8-item version of the scale, but this model had an unacceptable fit with the data (${\chi }^2$(18) = 121.49, CFI = 0.80, TLI = 0.72, RMSEA = 0.16, 90% CI [0.13, 0.18], SRMR = 0.078); only by adding several error covariance terms could this version of the scale be brought to acceptable levels of usability. We ran an additional CFA by adding a super-ordinate factor into the model. We did this firstly by merely adding an extra factor, and secondly by running a Schmid-Leiman (g-factor) analysis. Both of these models failed to converge properly and did not yield proper standard errors of the estimates (see our analysis script for further details: https://doi.org/10.6084/m9.figshare.14394446).

The principle of parsimony thus favors the 8-item, two-factor version presented in Fig. 3, below. While it is generally recommended that three items should minimally be used to estimate latent factors, it is also possible using only two items^39,40^, which conforms with the original scale design^12^. We also ran a parallel analysis on the 8-item version of the scale. The results (excluding the Kaiser criterion) indicated that there was only one factor. We also note that the predictive validity of the translated FSS did not change substantially depending on which version of the scale was used; though the 8-item scale had the most robust structure.

To measure internal reliability, we calculated the Cronbach’s alpha- and Tarkkonen’s rho values for the 6-, 8- and 10-item version of the scale; these values were 0.83, 0.84, 0.86 (Cronbach’s alphas), and 0.71, 0.75, and 0.76 (Tarkkonen’s rhos), respectively. Cronbach’s alpha values are sensitive to the number of items in the scale, but Tarkkonen’s rho corrects against this sensitivity. All scale versions show good reliability in terms of Cronbach’s alphas. However, in terms of Tarkkonen’s rho, the 6-item version is barely above the cutoff point of 0.71 (where signal-to-noise ratio > 1). The 8-item version is clearly above the cutoff point and not significantly different from the 10-item version (indicating that items 1 and 3 bring noise to the construct). Thus, reliability measures suggest that the 8-item FSS version should be preferred.

### External validity

In terms of external validity, the translated scale has already been used as a dependent variable in studies of a Flow-inducing game-like task^31–33^, which yielded sensible and strong findings (thus demonstrating the scale has good predictive properties in terms of both actual and self-reported behaviors).

**Figure 4:**
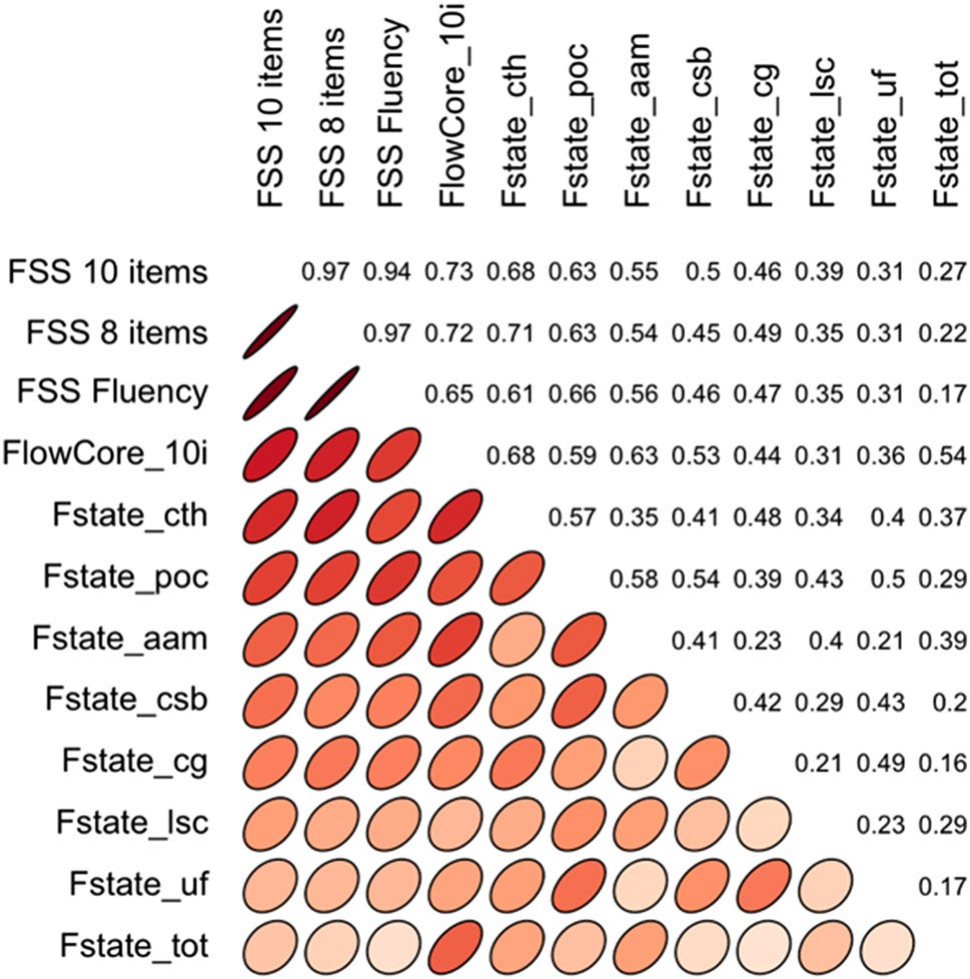
Sensitivity analysis (correlation matrix and visualization) for different versions of FSS. FSS = Flow Short Scale; FSS Fluency = 6 item FSS comprising only Fluency subscale; Fstate = Flow State Scale; cth = Concentration on task at hand; poc = Paradox of control; aam = Action-awareness merging; csb = Challenge-skill balance; cg = Clear goals; lsc = Loss of self-consciousness; uf = Unambiguous feedback; tot = Transformation of time. The ellipses and their colours depict the strength of the correlation (thinner and darker ellipse = stronger correlation).

We also ran a sensitivity analysis on 6-, 8- and 10-item versions of the FSS. The 6-item version (i.e. the Fluency subscale) excluded items 1, 3, 6 and 10, while the 8-item version excluded items 1 and 3. Sensitivity analysis in this context is a correlation matrix where all three versions of the FSS are correlated with the other Flow scales gathered at the same time: the Flow State and Core scales. The assessment is done qualitatively to estimate to what extent do correlations between the estimator variables fluctuate between the different scale candidates. As can be seen in Fig. 4, all the correlations between the 6-, 8-, and 10-item versions of FSS are fairly similar. Thus, sensitivity analysis further links the translated FSS with known scales with good demonstrated external validity.

### Face validity

**Figure 5:**
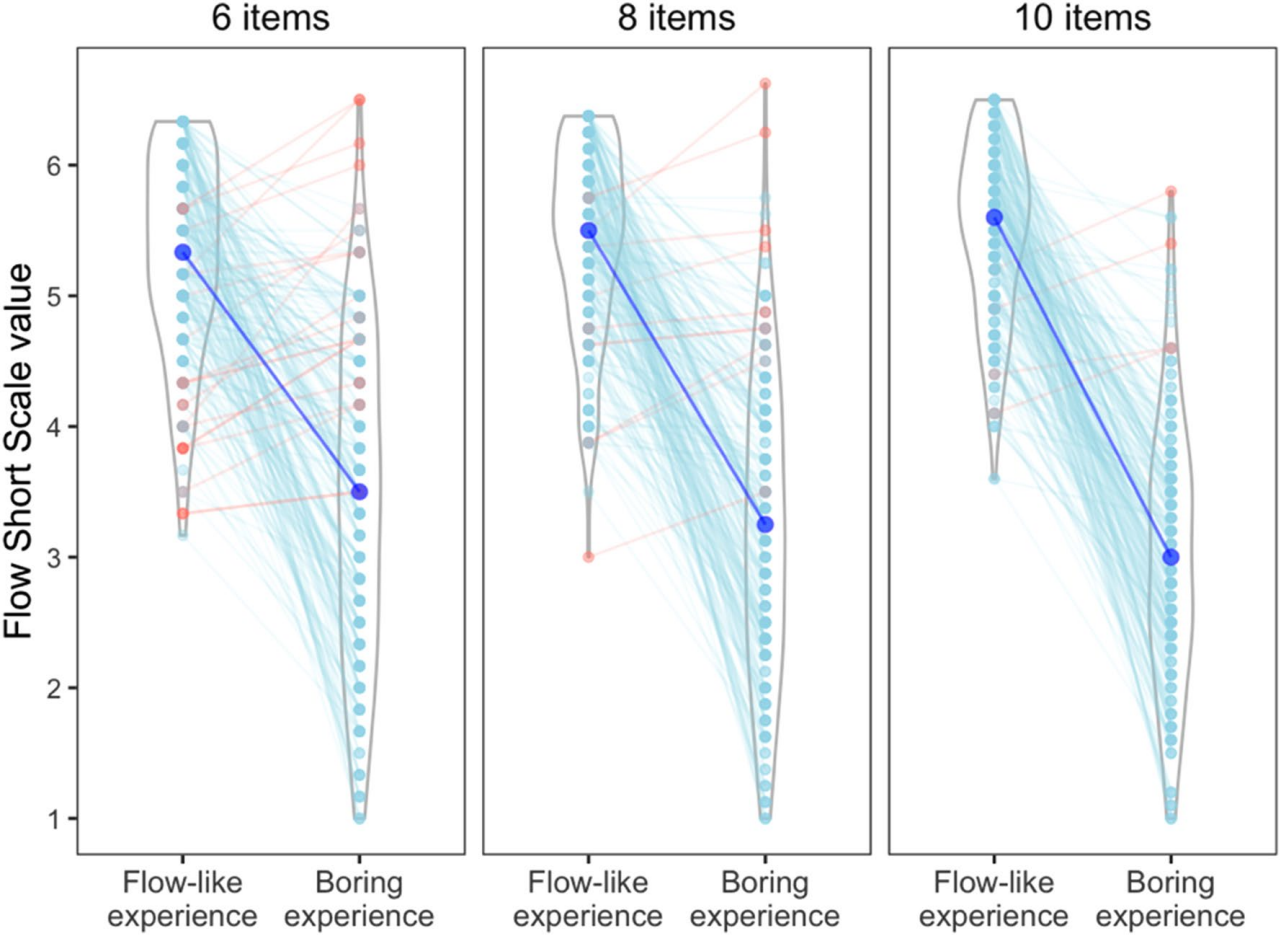
Comparison of Flow Short Scale (FSS) values between self-identified Flow-like and boring experiences, separately for 6-item-, 8-item-, and 10-item versions of FSS. Large blue dots represent median values, and the smaller dots represent observations from individuals. Red slopes indicate individuals who reported experiencing more Flow during their self-identified ‘boring’ experience, perhaps due to misunderstanding task instructions.

Finally, to evaluate how sensitive the FSS is to the difference between ‘boring’ and ‘Flow-like’ experiences, we ran a paired samples t-test comparing the two instruction conditions (as detailed in “Procedure” section). The test results provide clear evidence that the scale still functions as intended irrespective of how many items are included (6-item version: t(200) = 17.8, *p* < 0.001; 8-item version: t(200) = 22.6, *p* < 0.001; 10-item version: t(200) = 28.5, *p* < 0.001). Figure 5 illustrates the participant-wise condition differences for each FSS version: the 10-item version provides the clearest difference.

## Discussion

Our well-powered, comprehensively-analysed, scale validation study provides results that support the use of our Finnish translation of FSS with an 8-item, 2-factor version, which complements the state of the art of Flow measurement.

We also examined the 6-item version corresponding to only the ‘Fluency’ sub-scale, but ultimately, although this version does quite well in technical analysis, the choice should also be guided by a valid element of conservatism. Scales should develop with data incrementally, that is, not vary too wildly across studies (on vagaries of individual datasets). Another way to state it is that a scale is a model designed to predict a latent variable, and predictive models should always be hedged against overfitting to training data, to enhance generality and thus predictive power. On the other hand, the ten-item version clearly has problems with internal validity, but does quite well in tests of external and face validity. Ultimately, our recommendation to drop two items does not constrain the approach of any future users of the scale. Our work provides the full translated version of the FSS, along with empirical evidence for the ‘goodness’ of the three versions along several dimensions of validity, and users of the scale are thus well informed to choose their preferred approach.

Although the FSS represents the Flow experience as a unidimensional measure with 2 contributing conceptual factors, fluency and absorption, our analyses do not clearly support this conclusion. Do these two factors capture enough of the content of Flow, and are there other options? Our analyses support a two-factor solution, though with slightly different items than the original authors suggested. Generally, the items appeared to capture core aspects of Flow as evidenced by the scale’s correlation with other well-known flow-measures, and the scale’s overall ability to distinguish flow experiences from boring ones. However, the analyses do not constrain us to our chosen solution, and so (just as there was originally), there remains no empirical reason to separate fluency and absorption. This separation reflects a particular choice made by the original researchers developing the scale in German^26^. In their later study^12^, the same authors (with a sample of over 240 subjects) showed that the two-factor structure of fluency and absorption had internal consistency of $\alpha = 0.93$ and $\alpha = 0.78$ respectively, whereas a one-factor solution had internal consistency of $\alpha = 0.92$. Schüler^41^ reported similar reliability for these subscales, based on 57 subjects, with $\alpha = 0.87$ and $\alpha = 0.71$ respectively. They did not report on individual items, but the weaker consistency scores for absorption certainly agree with our analyses.

In comparing the 8-item, two-factor solution with the original 10-item scale, we must examine the dropped items also *conceptually*. For item 1 (“I feel just the right amount of challenge”), it is particularly notable that it failed to load on either factor. This replicates a result from a study which examined the psychometric properties of a Greek version of the FSS^25^. Csikszentmihaly^1^ did not conceptualize perceived challenge as a phenomenological element of flow experiences, but as a condition which fosters them. Thus, given that item 1 has neither conceptual nor empirical justification, we recommend to drop the item from the FSS. Item 3 describes the passing of time. This is problematic if the scale is to be used generally, in a wide variety of contexts with huge differences in the duration and time-constraints of the task. For instance, consider the study of athletic performance—how time is experienced is clearly different in a 100m dash that lasts 10s vs. an ultramarathon lasting up to several days—yet, arguably, Flow can be achieved in both. Finally, while the final 8-item solution contains a factor (absorption) with only 2 items (6 and 10), this is not a substantial concern (as noted above and by^39,40^). Items 6 and 10 are very well matched to the factor, both technically and conceptually, and all evidence supports the validity of this solution.

### Limitations

Despite sound psychometric properties, the FSS has the following limitations. The scale does not measure one of the key components of the Flow experience—its *autotelic* (i.e. enjoyable; intrinsically motivating) nature. From the beginning, Csikszentmihalyi explicitly conceptualized Flow as a form of enjoyment^42^. It was the enjoyable nature of Flow, and the positive implications this enjoyment had for motivation, he argued, that positioned it as a vehicle for skill development and personal growth (i.e., greater cognitive ‘complexity’)^7^. Given the centrality of enjoyment in Csikszentmihalyi’s conceptualization of Flow, it seems important for this aspect of the experience to be represented in the FSS.

A second limitation of the FSS (also common to other existing instruments) relates to its scale of measurement. Whereas Flow, as an ‘optimal’ state of consciousness, represents a discrete state, the FSS, like all Flow scales, operationalizes Flow as a continuous state, ranging from ‘low flow’ to ‘high flow’. Because level of intensity is built into the Flow construct, this results in conceptual imprecision. Kawabata and Evans^43^ proposed one way to deal with this inconsistency: by designating a ‘Flow cutoff’ when using Flow scales.

Ultimately, the choice to use FSS is well-motivated, since the alternatives share the same flaws and arguably also others. This shared fallibility should not constrain future work from seeking solutions to these problems.

Our study design had the following limitations. First, it is a single sample study—however, this problem is mitigated by the experimental design, which is uncommon in scale validation procedures and can be considered as a strength. While the sample size is sufficient, the sampling procedure could be improved to control representation of the general populace of Finnish speakers. We were fortunate to recruit a representative sample in gender, age, and education; the latter of which relates to language proficiency in standard written Finnish, i.e. the most important criterion for the scale translation validation.

### Future work

Our work, including the literature search described in the Introduction, highlights a major lacuna in the toolbox of optimal experience researchers globally. Firstly, as mentioned above, a recent review found that Flow was operationalized in 24 distinct ways^8^. Differences between these operationalizations were often considerable, so that the meaning of Flow sometimes changed substantially from one study to the next. Clearly, for our understanding of Flow as a single, coherent construct to progress, we need greater homogeneity in its measurement. Secondly, although reports on research are typically published in English, self-report data should ideally be gathered in participants’ native language. This is particularly important when working with constructs such as Flow that were derived from the semantic analysis of self-reports (originally, interviews conducted by Csikszentmihalyi and coworkers in the 1960s and 70s). Future work must test the properties of a multi-lingually translated Flow instrument with large independent samples.

Substantial resources and guidelines are invested to make validated translations available for, e.g. intelligence quotient instruments^9^. Although domain-specific re-validated translations by individual research groups are no doubt valuable, there is a clear need for a standardised general-purpose instrument available in multiple languages. Given the challenges described above, this need seems to require a consensus-driven international consortium project, which would move us closer to harmonising the field of Flow research. Such a project would also address a limitation of our study: the domain of generalizability is not clear, because we do not know whether the Flow measurement problems are language specific or construct specific (notwithstanding the convergence on item 1 with^25^).

Future work on such a universally valid Flow-scale needs to pay attention to cross-cultural observations of invariance in the factor-loadings and intercepts of the scale. It is important to observe whether the cross-culturally translated versions of the Flow scale differ in the sensitivity of the items, or simply whether the items have a lower base-line in detecting Flow experiences. Such invariance analysis would also weed out more fundamental questions as are there differences in how Flow-inducing or ‘flow-positive’ different cultures potentially are. As a hypothetical example, cultures that are familiar with ecstatic dancing or trance-inducing techniques might be more open to Flow experiences compared to cultures that do not incorporate such techniques.

Further investigations, with larger samples and cross-cultural comparison, could also utilize the Schmid-Leiman factor analysis to investigate whether there is a *g*-factor of Flow, that would argue more strongly for a unidimensional solution^44^. If no *g*-factor for Flow is found in larger samples, this would also help settle the long-standing dispute on how Flow should be conceptualized. As an extension to such analyses, if it turns out that there are meaningful multidimensional structures to the Flow scale, multidimensional item response theory analyses could be used. In such an analysis, single items could provide meaningful cross-loadings on several latent factors simultaneously. However, if such items would then be used in experimental setups, it is not clear how they should be used. Nonetheless, it seems that a thorough psychometric project shared with several data-collection locations is called for in the near-future horizon of Flow-scale development research.

### Conclusion

We have presented a psychometric validation of the Flow Short Scale in a Finnish language translation. Considering the current fleet of validated Flow scales, we believe the FSS represents one of the better options out there. The FSS is short, consisting of only 10 items, and such brevity is desirable given that Flow is often measured in repeated-measures designs. Compared to the Flow State Scale (the most commonly used Flow scale), the FSS conflates the experience of Flow with the conditions of Flow to a lesser extent^8^. Finally, the FSS is commonly used in Flow research, which means that its results can be meaningfully compared across a number of studies. It is also important to note that, although our original experimental studies used wording focused on game play^31,33^, in this validation study we changed the wording to focus on a generic task activity. Thus, the validated Finnish FSS is viable for use in a variety of domains beyond game-play.

This instrument will provide value to a range of cutting edge Finnish research on optimal experience and peak performance in, for example, education, sports, music, work life, human-computer interaction, and military domains. In turn, this will translate to added benefits for society in Finland and beyond, exemplified by the world-leading Finnish education system.

## Data Availability

The R syntax for all analyses reported here, and the datasets generated and/or analysed during the current study, are available in the same repository, https://doi.org/10.6084/m9.figshare.14394446.
